# Neutron star merger remnants

**DOI:** 10.1007/s10714-020-02752-5

**Published:** 2020-11-10

**Authors:** Sebastiano Bernuzzi

**Affiliations:** Theoretisch-Physikalisches Institut, Friedrich-Schiller-Universität Jena, Max-Wien-Platz 1, 07743 Jena, Germany

**Keywords:** Binary neutron star, Mergers, Remnants, Gravitational waves, Numerical relativity

## Abstract

Binary neutron star mergers observations are a unique way to constrain fundamental physics and astrophysics at the extreme. The interpretation of gravitational-wave events and their electromagnetic counterparts crucially relies on general-relativistic models of the merger remnants. Quantitative models can be obtained only by means of numerical relativity simulations in $3+1$ dimensions including detailed input physics for the nuclear matter, electromagnetic and weak interactions. This review summarizes the current understanding of merger remnants focusing on some of the aspects that are relevant for multimessenger observations.

## Introduction

The gravitational-wave GW170817 is compatible with a binary neutron star (BNS) inspiral of chirp mass $1.186(1)M_{\odot }$ [1–3]. Significant signal-to-noise ratio (SNR) is found in the frequency range 30–600 Hz, roughly corresponding to the last thousand orbits to merger for an equal-mass binary with canonical mass $M\sim 2.8\,M_{\odot }$. The matched-filtering analysis of GW170817 with tidal waveform templates provides us with an estimate of the reduced tidal parameter that is distributed around $\tilde{\varLambda }\sim 300$ and smaller than ${\sim }800$ [4–7]. LIGO-Virgo searches for short (${\lesssim }1\,$s), intermediate (${\lesssim }500\,$s) and long (days) postmerger transients from a neutron star (NS) remnant resulted in upper limits of more than one order of magnitude larger than those predicted by basic models of quasi-periodic sources [8–12]. Hence, the LIGO-Virgo detectors’ sensitivity was not sufficient to detect a signal from the merger phase and the remnant, which lie in the kiloHertz range [13]. A similar conclusion holds for the second BNS event, GW190425, that was detected at lower SNR than GW170817 [14]. GW190425 has a chirp mass of $1.44(2)\,M_{\odot }$ and it is associated to the heaviest BNS source known to date.^1^

In absence of a GW detection, the merger remnant can be inferred from the binary properties and from the interpretation of the electromagnetic counterparts based on the theoretical predictions given by numerical relativity (NR) simulations. The latter are the only method available to determine the merger outcome and to compute the GW signals from the remnants. This review summarizes the current understanding of merger remnants as determined by NR simulations during the last 20 years.^2^ The presented results are key for the interpretation of future observations of multimessenger signals from BNS mergers.

Current numerical relativity methods applied to quasicircular mergers allow us to simulate tens of orbits before merger and the early postmerger phase for a timescale of several dynamical periods. Inspiralling NSs are well-described by zero-temperature matter in beta-equilibrium with maximum density about twice the nuclear saturation density $\rho _{\mathrm{NS}}\sim 2$–$3\rho _0$ ($\rho _0\simeq 2.3\times 10^{14}$ $\text {g cm}^{-3}$). Electromagnetic fields are not expected to significantly affect the mass dynamics [16, 17]. Thus, general relativistic simulations with perfect fluid matter are believed to faithfully model the orbital phase. The inspiral dynamics can be characterized in terms of the binary masses (and spins), and the tidal polarizability parameters, as described in Sect. 2. At the end of the inspiral, about 3–4% of the initial gravitational mass is radiated in GWs and the binary merges at typical GW frequencies ${\sim }$1–2 kHz.

Binary NS mergers result in the formation of a compact central object, either a NS or a black hole (BH), eventually surrounded by an accretion disc [18–21]. The remnant can be characterized in first approximation by the NS masses and by the softness of the (unknown) zero-temperature equation of state (EOS), in particular by the maximum sustainable mass, $M_\mathrm {max}^\mathrm {TOV}$ [22, 23]. Binary remnants with total mass significantly larger than $M_\mathrm {max}^\mathrm {TOV}$ cannot be sustained by the EOS pressure and by the centrifugal support of their rotations. Thus, the remnant promptly collapses to a BH during its formation. A precise definition of prompt BH collapse and the phenomenology inferred from the simulations are discussed in Sect. 3.

If the remnant does not promptly collapse, its early evolution is driven by GW emission and characterized by a luminous GW transient emitted at frequencies ${\sim }$2–4 kHz [19, 24–28]. Matter in NS remnants is compressed and heated up to extreme densities and temperatures, and the baryon mass density can reach $\rho _\mathrm{rem}\sim 1.5-2\rho _{\mathrm{NS}}\sim 3-6\rho _0$ and temperatures $\gtrsim 50$ MeV [29, 30]. The NS remnant can either collapse to BH after a “short life” on the dynamical timescale determined by its rotational period, or settle to an axisymmetric equilibrium configuration on longer timescales. The black holes that can be produced in BNS mergers are discussed in Sect. 4.

After the GW-driven, dynamical phase, the angular momentum of the NS remnant at formation is well above the Keplerian (mass-shedding) limit of an equilibrium zero-temperature beta-equilibrated rigidly-rotating configuration with the same baryon mass [31]. Also, the remnant has gravitational mass in excess of those equilibrium configurations. Thus, it is far from equilibrium and its long-term evolution is determined by the energy and angular momentum evolution due to magnetohydrodynamics and weak interactions in the fluid, as well as GW emission [32–35]. Neutron-star remnants and their evolutionary phases are discussed in Sect. 5.

A key dynamical feature for GW counterparts is the formation of remnant discs [36–40]. Remnant discs of masses ${\sim }0.1\,M_{\odot }$ can form if the matter acquires sufficient rotational support during merger. The initial composition and extension of a remnant disc is dependent on whether the central object is a NS or a BH. The disc evolution starts with a phase of rapid accretion, but is afterwards determined by a combination of the gravitational pull, the neutrino cooling and the expansion due to viscous processes and magnetic field stresses [32, 41–45]. The properties of remnant discs are discussed in Sect. 6.

During merger, a mass ${\sim }10^{-4}$–$10^{-2}\,M_{\odot }$ of neutron rich material is expelled on dynamical timescales [33, 46–49]. The remnant can unbind an even larger amount of material by winds powered by different mechanisms [45, 50–53]. These ejection mechanisms and NR-based estimates of ejecta masses and composition are reviewed in Sect. 7. Mass ejecta from mergers are a key astrophysical site for heavy-element production via the r-process [49, 54–58]. The observational imprint of r-process element production is the kilonova electromagnetic transient, that was observed for the first time as the counterpart of GW170817. Because of their quasi-isotropic character, kilonovae are considered to be the most promising EM counterpart for future GW events [59–63].

“Appendix A” summarizes the main input physics and numerical techniques employed for the NR simulations.

Geometric units $G=c=1$ are used if units are not explicitely indicated.

**Fig. 1:**
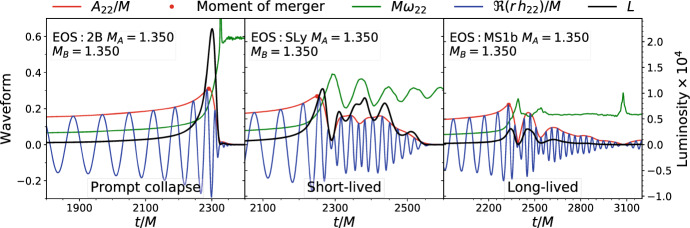
Gravitational-waveforms for representative equal-mass irrotational mergers. The figure shows the evolution of amplitude, frequency and real part of the (2, 2) multipole of the GW strain and luminosity. From left to right: prompt collapse, short-lived, and long-lived stable remnant. Figure adapted from [64]

## Merger dynamics

The inspiral BNS dynamics differ from those of the binary black hole because of the tidal interactions between the NSs. Tidal interactions in the post-Newtonian formalism for self-gravitating and deformable bodies are calculated using a multi-chart approach, in which the tidal response of a NS due to the external gravitational field of the companion (the inner problem) is matched to an outer problem for the description of the orbital dynamics and radiation [65–69]. In the local frame of body *A*, the mass multipole moments $M^A_L$ 3 are related to the external gravitoelectric moments $G^A_L$ by $M^A_L = \mu ^A_\ell G_L^A$ where $L=i_1...i_\ell$ is a multi-index. Analogously to the electric polarizability of a charge distribution, the coefficients $\mu _\ell$ quantify the distortion of the mass distribution due to the external field. They are often substituted by the dimensionless relativistic Love numbers obtained by normalizing with the appropriate power of the NS radius,

1 $$\begin{aligned} k^A_\ell := \frac{(2\ell -1)!!}{2}\frac{G\mu ^A_\ell }{R^{2\ell +1}_A} \, . \end{aligned}$$

The practical calculation of the Love numbers reduces to the solution of stationary perturbations of spherical relativistic stars [65, 70–72]. The Love numbers are thus dependent on the EOS employed for constructing the equilibrium NS and on the NS compactness, $C_\mathrm {A}=GM_\mathrm {A}/(c^2R_\mathrm {A})$. In the following we will make use exclusively of the quadrupolar *tidal polarizability parameters* defined as [69, 73]

2 $$\begin{aligned} \varLambda _A := \frac{2}{3} k^A_2 \left( \frac{GM_A}{R_Ac^2}\right) ^{5} \, . \end{aligned}$$

The two-body relative dynamics in the weak field regime is described by the Newtonian Hamiltonian with a tidal term in the potential [4, 69]

3 $$\begin{aligned} H \simeq \frac{\mu }{2} p^2 + \frac{\mu }{2} \left( -\frac{2GM}{c^2 r} + ... - \frac{\kappa _2^T}{r^6} \right) \, , \end{aligned}$$

where $\mu$ is the reduced mass of the binary. The tidal coupling constant $\kappa _2^T$ is defined as

4 $$\begin{aligned} \kappa _2^\mathrm {T} := \dfrac{3}{2} \left[ \varLambda _2^\mathrm {A} \left( \frac{M_\mathrm {A}}{M}\right) ^4 \frac{M_{B}}{M} + (A\leftrightarrow B) \right] \, , \end{aligned}$$

and parametrizes at leading (Newtonian) order the tidal interactions in the binary. The formula above indicates that tidal interactions are attractive and short-range.^4^ The effect of tidal interactions in the inspiral is illustrated by the (modified) Kepler law [69],

5 $$\begin{aligned} \varOmega ^2 r^3 = GM\left[ 1+ 12\frac{M_A}{M_B}\frac{R_A^5}{r^5}k^A_2 + (A\leftrightarrow B)\right] \, . \end{aligned}$$

At a given radius the frequency is higher if the tidal interactions are present. Thus, the motion is accelerated by tidal effects and the system merges earlier and at a lower frequency. We shall see that, while the details of tidal interactions during merger can be quantified only by general relativistic hydrodynamical simulations, these basic results are key to characterize the merger data from the simulations. Note that the reduced tidal parameter [5]

6 $$\begin{aligned} {\tilde{\varLambda }} := \frac{16}{13} \frac{(M_\mathrm {A} + 12 M_\mathrm {B}) M_\mathrm {A}^4}{M^5}\varLambda _\mathrm {A} + (A\leftrightarrow B)\ , \end{aligned}$$

is also used to parametrize tides at leading order in place of $\kappa ^\mathrm {T}_2$. Although not the same quantity, ${\tilde{\varLambda }}$ and $\kappa ^\mathrm {T}_2$ will be used here for the same purposes. The ranges for BNSs are $\kappa ^\mathrm {T}_2\approx (20,500)$ and ${\tilde{\varLambda }}\approx (50,2000)$. Softer EOS, larger masses and higher mass-ratios result in smaller values of $\kappa ^\mathrm {T}_2$ (or ${\tilde{\varLambda }}$). In what follows we discuss an effective characterization of the merger properties relevant for the later discussion on the merger remnant.

**Fig. 2:**
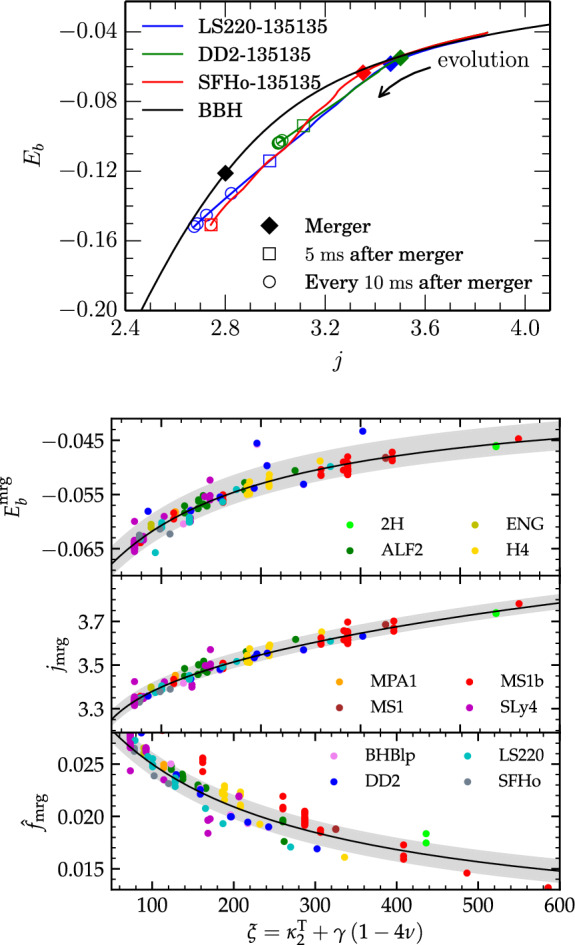
Energetics of BNS mergers and quasiuniversal (EOS-independent) relations at the moment of merger. Top: Evolution of the reduced binding energy and angular momentum (see Eqs. 8–9) for representative models and comparison to the binary black hole case. The moment of merger is marked by a squared black bullet, subsequent times are marked with empty bullets. Figure from [74]. Bottom: Reduced binding energy, and angular momentum and GW frequency at the moment of merger as a function of the $\xi (\kappa ^\mathrm {T}_2,\nu )$ parameter (Eq. (11)) from the CoRe database of simulations. Note that the frequency is rescaled by the mass to give a dimensionless quantity, $\hat{f}_{\mathrm{mrg}}=Mf_{\mathrm{mrg}}$. Gray bands represent the fit 90% confidence region. Figure adapted from [75]

The BNS dynamics in numerical relativity are usually studied by considering the gravitational radiation computed during the simulations. The latter is extracted from coordinate spheres at finite radii *R* and extrapolated to null-infinity. Simulations resolve the first modes of the multipolar decomposition,

7 $$\begin{aligned} R\left( h_+ - i h_\times \right) = \sum _{\ell =2}^{\infty }\sum _{m=-\ell }^\ell h_{\ell m}(t)\; {}^{-2}Y_{\ell m}(\theta ,\varphi ) \approx h_{22}(t)\; {}^{-2}Y_{22}(\theta ,\varphi ) + c.c. \, , \end{aligned}$$

where ${}^{-2}Y_{\ell m}$ are the spin-weighted $s=-2$ spherical harmonics. Examples of circular merger gravitational-waves are shown in Fig. 1 together with the instantaneous GW frequency and luminosity. All the waveforms show the chirp behaviour, predicted by the post-Newtonian formalism, that terminates at a characteristic amplitude peak, the time of which is sometimes referred to as the *moment of merger* (and distinguished from the merger process). The moment of merger marks the end of the chirp signal. Note that the luminosity peak is delayed with respect to the amplitude peak.

A gauge invariant way to characterize the BNS dynamics using simulation data is to consider the reduced binding energy and angular momentum of the binary, computed as [76, 77]

8 $$\begin{aligned} E_b&= - \dfrac{M - \varDelta E_\mathrm {GW}}{\mu } = \dfrac{\left( M_\mathrm {ADM} - \varDelta {\mathcal {E}}_\mathrm {GW}\right) - M}{\mu } \end{aligned}$$

9 $$\begin{aligned} j&= \dfrac{J_\mathrm {ADM}-\varDelta {\mathcal {J}}_\mathrm {GW}}{M\mu } \, . \end{aligned}$$

Above, *M* is the binary mass and $\varDelta {\mathcal {E}}_\mathrm {GW}$ and $\varDelta {\mathcal {J}}_\mathrm {GW}$ are the radiated energy and angular momentum computed from the multipoles $h_{\ell m}$ during a simulation. The total binding energy $\varDelta E_\mathrm {GW}$ and the binary’s angular momentum are computed from $\varDelta {\mathcal {E}}_\mathrm {GW}$ and $\varDelta {\mathcal {J}}_\mathrm {GW}$ by subtracting the contribution of the Arnowitt–Deser–Misner (ADM) energy and angular momentum of the initial data. During the evolution, a binary emits energy and angular momentum and both *j* and $E_b$ decrease from their initial values, as shown in the top panel of Fig. 2. A dynamical frequency can be defined as

10 $$\begin{aligned} M\varOmega = \frac{\partial E_b}{\partial j}\, \end{aligned}$$

using the Hamilton–Jacobi equations. This frequency corresponds to half the GW frequency $\omega _{22}=-\mathfrak {I}{({\dot{h}}_{22}/h_{22})}$ of the dominant (2, 2) mode, and thus can be identified with the binary’s orbital frequency during the inspiral [76]. However, the validity of Eq. (10) is not restricted to the inspiral, and $\varOmega$ can be used to characterize also the postmerger evolution. Simulations have shown that the instantaneous frequency of the postmerger waveform is also $\omega _{22}\approx 2\varOmega$ [28].

As suggested by Fig. 1, the GW quantities (frequency, peak amplitude and luminosity) and thus also the energetics are very dependent on the binary mass and mass ratio as well as on the NSs’ EOS and spins. However, using the analytical estimates presented above it is possible to describe all the numerical data in simple terms. At sufficiently high frequencies the short range tides significantly contribute to the binary interaction energy^5^ and the key dynamical quantities and the GW are functions of the tidal parameter [78]. For example, the properties of every simulated equal-mass binary at the moment of merger are very well captured by $\kappa ^\mathrm {T}_2$ solely. The fact that the latter parameter encodes to a very good accuracy the EOS is sometimes referred to as *quasiuniversality*; relations like $f(\kappa ^\mathrm {T}_2)$ are called EOS-independent or EOS-insensitive relations. Mass-ratio effects up to $q=M_A/M_B\sim 2$ can be described by further considering the parametrization

11 $$\begin{aligned} \xi = \kappa _2^T + \gamma (1-4 \nu ) \, , \end{aligned}$$

where $\nu =\mu M\in [0,1/4]$ and $\gamma$ is a fitting parameter [75].

Figure 2 shows the robusteness of this description for a large number of irrotational BNS simulations. More compact (small $\kappa ^\mathrm {T}_2$) and more massive binaries emit more energy, as expected. A fiducial equal-mass merger emits about 3–4% of the mass in GWs by the end of the chirp phase (for irrotational binaries). The angular momentum of the system at merger is larger the less compact the binary is and the larger (smaller) the mass ratio *q* ($\nu$) is. In other terms, binaries with NSs with large radii merge at larger separations. The GW merger frequency can be fit to a simple function of $\xi$

12 $$\begin{aligned} f^\mathrm {mrg}_\mathrm {GW} \simeq 2.405 \left( \frac{1 + 1.307\, \cdot 10^{-3} \xi }{1 + 5.001\,\cdot 10^{-3} \xi }\right) \left( \frac{M}{2.8 M_{\odot }}\right) \ \mathrm{kHz} \, , \end{aligned}$$

with $\gamma \simeq 3200$. Similar relations exists for all the relevant dynamical quantities, such as the binding energy, the angular momentum, or the GW luminosity at merger [64, 75, 79]. The effects due to the NS rotation can also be included in this picture. The largest spin effect is given by spin-orbit interactions that depend, to first approximation, on the magnitude and sign of the projection of the spin along the orbital angular momentum, $S_z$. For small spins the effect is linear in spin and, for example, the angular momentum at merger is $j_S \approx j_0 \pm S_z/M\mu$ [79, 80].

We shall see in the following that $\kappa ^\mathrm {T}_2$ (or ${\tilde{\varLambda }}$) is a useful “order parameter” also for some properties of the remnant. While there are no binary dynamics in this case, the remnant quantities at early times are largely determined by the conditions at merger.

**Fig. 3:**
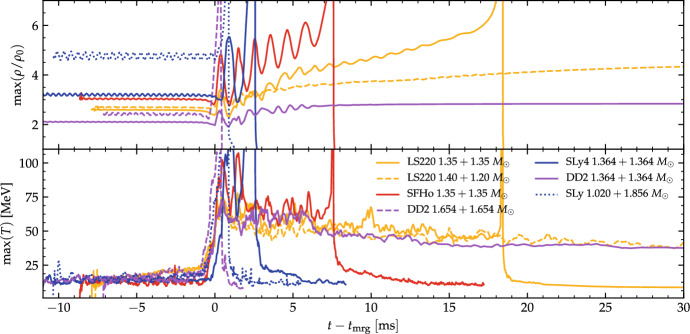
Evolution of the maximum density and temperature during mergers of representative binaries. The legend indicates the total mass. The DD2 $1.654+1.654\ M_{\odot }$ model is an example of prompt collapse; SLy $1.020+1.856\ M_{\odot }$ is an example of accretion induced prompt collapse. In each case the maximum density monotonically increases to the collapse. SLy $1.364+1.364\ M_{\odot }$, SFHo and LS220 $1.35+1.35\ M_{\odot }$ are short-lived remnants collapsing to a BH at different times. Note that the oscillations in the maximum density are related to the NS cores bouncing and are correlated to the temperature peaks. LS220 $1.20+1.40\ M_{\odot }$ shows that moderate mass ratios can increase the lifetime of the remnant (compare to the LS220 equal mass case). DD2 $1.364+1.364\ M_{\odot }$ is an example of long-lived remnant surviving for more than 100 ms. Figure adapted from [30, 33, 81]

## Prompt collapse

Prompt BH collapse mergers can be defined as those mergers in which the NS cores collision has no bounce, but instead the remnant immediately collapses at formation (See also the discussion in Sect. 5). Prompt collapse happens within ${\sim }$1–2 ms from the moment of merger and can be identified by the maximum density monotonically increasing to the collapse. Two examples of prompt collapse mergers are shown in Fig. 3, for which the maximum density increases beyond $6\rho _0$ at $t\approx t_{\mathrm{mrg}}$. This definition of prompt collapse implies negligible shocked dynamical ejecta because the bulk of this mass ejection comes precisely from the (first) core bounce [33]; we shall expand on this point in Sect. 7. An example of a merger waveform for a prompt collapse is shown in the first panel of Fig. 1. The signal’s amplitude goes rapidly to zero while the GW frequency increases to the BH quasi-normal-mode frequencies. At this point in the simulation, an apparent horizon has formed and it has reached an approximately stationary state.

Numerical-relativity simulations predict that circular and equal-mass BNS mergers will be followed by a prompt collapse to a BH, if the total gravitational mass *M* of the binary exceeds a threshold mass, given by [22, 23]

13 $$\begin{aligned} M_\mathrm {pc}= k_\mathrm {pc}M_\mathrm {max}^\mathrm {TOV}\, , \end{aligned}$$

where $k_\mathrm {pc}$ depends on the unknown EOS. Current simulations of irrotational, $q\sim 1$ BNSs spanning a sample of 18 hadronic EOS and comparable masses find that $1.3 \lesssim k_\mathrm {pc}\lesssim 1.7$ [22, 23, 64, 82, 83]. For these data $k_\mathrm {pc}$ shows an approximately EOS-insensitive linear behaviour in the compactness *C* (or in the radius) of a reference nonrotating NS at equilibrium [23]. For example, using the extended set of data from [22, 23, 83], and choosing the maximum NS compactness as a reference one finds the best fit [84]

14 $$\begin{aligned} k_\mathrm {pc}(C_\mathrm {max}) = -(3.29\pm 0.23) \, C_\mathrm {max} + (2.392\pm 0.064) \, . \end{aligned}$$

Note that under the hypothesis that the merger did not promptly form a BH, the inversion of Eq. (13) leads to a bound on the maximum NS mass. This argument can be used to estimate the maximum NS mass after GW170817, by interpreting the GW counterpart as evidence for a NS remnant [85–88].

An alternative model for the prompt collapse threshold based on NR data is based on the tidal parameter $\kappa ^\mathrm {T}_2$ [64]. An analysis of comparable-mass data of the CoRe collaboration finds that all the reported prompt collapse mergers are captured by the condition^6^

16 $$\begin{aligned} \kappa ^\mathrm {T}_2 < \kappa ^{\mathrm{T}}_\mathrm {pc} \approx 80\pm 40 \, , \end{aligned}$$

which is a quasiuniversal relation. For equal-mass BNSs, $\kappa ^\mathrm {T}_2$ can be interpreted as a measure of the binary compactness with more compact binaries leading to earlier BH formation. Note that Eq. (16), differently from Eq. (13), contains a dependence on the mass ratio. Improved phenomenological descriptions of the collapse threshold can be obtained by parametrizing the threshold using both the maximum mass and the tidal parameter $\kappa ^\mathrm {T}_2$ [89].

The above prompt collapse models are valid for comparable masses and irrotational (no NS spin) mergers. For a given total mass, moderate mass ratios can extend the remnant lifetime with respect to an equal mass BNS because of the less violent fusion of the NS cores and a partial tidal disruption that distributes angular momentum at larger radii in the remnant [23]. If the total mass is sufficiently large, the primary NS can be close to $M_\mathrm {max}^\mathrm {TOV}$ and the material accreting from the (partial) tidal disruption of the companion can favour a prompt collapse. Moreover, spin-orbit interactions have repulsive or attractive character depending on the sign of the spin projection along the orbital angular momentum (spins aligned or antialigned). Hence, they can either increase (or decrease) the angular momentum support of the remnant and delay (anticipate) BH collapse [80, 90, 91].

The prompt collapse models above indeed fail for large mass ratios $q\sim 1.5$–2 [81]. In BNSs with increasing mass ratios and fixed chirp masses, the companion NS undergoes a progressively more significant tidal disruption. For a sufficiently soft EOS, the collapse in these mergers is triggered by the accretion of the companion onto the massive primary star. This “accretion-induced prompt collapse” scenario should be always present after a critical mass ratio in connection to the maximum NS mass. A rough estimate of the threshold is given by modiyfing Eq. (13) as $M_\mathrm {pc}(\nu )\sim M_\mathrm {pc}\cdot (4\nu )^{3/5}$, and it indicates that extreme mass ratios favour prompt collapse.

A systematic numerical-relativity investigation of the prompt collapse threshold varying the input EOS models (for example also considering hyperons and phase transitions [89, 92–95]), masses, mass ratio and spin is presently missing but rather urgent for a quantitative understanding of the merger dynamics. Related to this, it remains challenging to construct an EOS-insensitive (universal) relation for robustly determining the prompt collapse from binary properties.

## Remnant black holes

Black holes produced by the collapse of irrotational binary merger remnants (either prompt collapse or short-lived) are found with dimensionless spin [74, 80, 81, 90, 96, 97]

17 $$\begin{aligned} 0.6 \lesssim a_\mathrm {BH} \lesssim 0.875 \, . \end{aligned}$$

This interval can be expected from the merger quasiuniversal relations presented in Sect. 2. The relations for $E_b^\mathrm {mrg}(\kappa ^\mathrm {T}_2)$ and $j_\mathrm {mrg}(\kappa ^\mathrm {T}_2)$ at the moment of merger give upper limits for the BH mass and spin

18 $$\begin{aligned} M_{\mathrm{BH}}< E_b^\mathrm {mrg}\nu M \ \ \text{ and } \ \ a_{\mathrm{BH}} < j_\mathrm {mrg}\nu \, , \end{aligned}$$

assuming the remnant would instantaneously collapse to a BH without GW emission nor remnant disc/ejecta. The reduced angular momentum range in Fig. 2 is $3.2\lesssim j_\mathrm {mrg}\lesssim 3.8$, with smaller values obtained for smaller $\kappa ^\mathrm {T}_2$. Binaries with $\kappa ^\mathrm {T}_2\gtrsim 250$ have stiff EOS and the remnants are either short or long-lived. Such remnants emit in GWs at least the same amount of binding energy that they posses at merger (Fig. 2, top panel), hence one can focus on binaries that collapse promptly with $\kappa ^\mathrm {T}_2<120$ (Eq. (16)) and obtain $a_{\mathrm{BH}}<0.875$ for equal-mass BNS ($\nu =1/4$).

The largest BH spins, $a_{\mathrm{BH}}\sim 0.8$, are obtained for equal-mass prompt collapse mergers. Note that, in this case, the postmerger GW luminosity is comparable to that of the moment of merger and that very light discs are formed. For large mass ratios the angular momentum at merger is distributed in a massive accretion disc and the BH spin is below the upper limit. Black holes formed by the collapse of short-lived NS remnants have typically smaller spins than those produced in prompt collapses (for a given mass), because their postmerger GW emission is significant (as will be discussed in Sect. 5) and they are surrounded by massive accretion discs.

Remnant BHs can spin up due to the disc accretion and, in principle, can reach almost maximal spins [98, 99]. In practice however, Keplerian discs in merger remnants are too light to significantly spin-up the BH. Moreover, ordered poloidal magnetic fields between the disc and the horizon can transport angular momentum outward into the bulk of the disc and even arrest the accretion [100–102]. The disc accretion can be further modified by the angular momentum losses due to winds on the same timescales [103–105], and the launch of a jet might also spindown the BH [106]. The evolution of the remnant BH on timescales of seconds is an open question related to the accretion disc dynamics, that will be further discussed in Sect. 6.

The upper limits on the BH rotation inferred from NR simulations should be considered in models of electromagnetic counterparts. For example, in short-gamma-ray burst models (SGRBs) the energy deposition by neutrino pair-annihilation depends strongly on the BH spin [107]. For fixed accretion rate, the energy deposition by neutrinos from a disk accreting onto a BH with $a_{\mathrm{BH}}=0.7$ can be up to a factor 100 times smaller than for a disk feeding a maximally spinning BH. On the other hand, $a_\mathrm {BH}$ does not significantly constrain SGRB models invoking magnetic mechanisms, which can easily account for the required energies even in the absence of a highly spinning BH [108]. Note that in the Penrose/Blandford–Znajek mechanism the BH rotational energy is extracted at a rate proportional to $a_\mathrm {BH}^2$ at leading order in spin [102, 109]. We refer the reader to recent reviews for a complete discussion on the accretion flow onto BHs and its connection to SGRBs [110–112].

## Remnant neutron stars

The observations of pulsars with masses ${\sim }2M_{\odot }$ [113, 114] constrain EOS models to support maximum masses larger than ${\sim }2M_{\odot }$. In this scenario, a likely outcome of a fiducial $M\sim 2.8M_{\odot }$ merger is a NS remnant, e.g. [22]. The properties and evolution of these NS remnants discussed here below are subject of intense research and closely linked to observations of kiloHertz GW and mergers’ counterparts.

It is customary to define short-lived NS remnants as those collapsing on the timescale of their rotational periods (tens of milliseconds), and long-lived remnants those collapsing on significantly longer timescales. Often, short-lived remnants are referred to as hypermassive NSs (HMNS), while long-lived remnants are referred to as either supramassive NSs (SMNS) or massive NSs (MNS). Throughout this work we do not use the names HMNS and SMNS for merger remnants 7 since these names refer to general-relativistic zero-temperature axisymmetric equilibrium configurations, but merger remnants are not cold equilibria. In particular, a HMNS is defined as a differentially rotating NS at equilbrium with mass above the rigidly rotating limit [115], while a SMNS (MNS) is a rigidly rotating NS at equilibrium with rest mass larger (smaller) than the nonrotating equilibrium limit $M_\mathrm {max}^\mathrm {TOV}$ [116, 117].

The evolution of the remnant can be approximately separated into an early (dynamical) GW-driven phase and a secular phase that is (initially) driven by viscous magnetohydrodynamics processes and neutrino cooling. The fate of the remnant is determined by a complex interplay of gravitational, nuclear, weak and electromagnetic interations that often act on comparable timescales.

*Dynamical (GW-driven) phase.* At formation, NS remnants are very dynamical. The maximum density and temperature increase immediately after merger as a consequence of matter compression and the NS cores bounce several times, e.g., [30]. The more massive and compact the binary, the faster and the more violent the dynamics are. Despite the large relative collision speed, the speed of sound at densities $\rho \gtrsim \rho _0$ is $c_s \gtrsim 0.2$c and prevents the formation of hydrodynamical shocks inside the cores. Only at the surface of the NSs pressure waves can steepen into shock waves which accelerate matter at the edge of the remnant up to mildly-relativistic speeds. Thus, matter inside the cores remains cold ($T \lesssim 10\,\mathrm{MeV}$) and, while the densest regions of the cores rotate and fuse, the compressed matter at the contact interface is pushed outwards. Matter moving outwards reaches temperatures up to $T\sim 70{-}110~\mathrm{MeV}$ and forms a pair of co-rotating hot spots displaced by an angle of ${\sim } \pi /2$ with respect to the densest cores, e.g. [118]. The bound matter expelled from the center forms a disc which is fed by the central remnant with hot and outgoing density spiral waves streaming from the central region (see also the discussion in Sect. 6.)

The high temperatures in the remnant determine high neutrino production and an early burst in neutrino luminosity reaching ${\sim }10^{52-53}$ erg/s, e.g. [29, 119–121]. Simulations including neutrino transport predict the mean neutrino energies at infinity $E_{\nu _e} (\sim 10~\mathrm{MeV}) \lesssim E_{\bar{\nu }_e} (\sim 15~\mathrm{MeV}) \lesssim E_{\nu _{\mu ,\tau }} (\sim 20~\mathrm{MeV})$, with more massive binaries and softer EOS resulting in higher mean energies [29, 122, 123]. Due to the strong dependence of the cross-sections on the incoming neutrino energy, neutrinos with different energies decouple from matter in very different regions. At the average energies, $\nu _e$ and $\bar{\nu }_e$ decouple at densities between a few and several times $10^{11}\mathrm{g~cm^{-3}}$, respectively. Low energy neutrinos decouple at around $10^{13} \mathrm{g~cm^{-3}}$ along spheroidal neutrino decoupling surfaces [123, 124]. Because free neutrons are abundant, the absorption opacities for $\nu _e$ are larger than those for $\bar{\nu }_e$, while pair processes, responsible for keeping $\nu _{\mu ,\tau }$ and their antiparticles in equilibrium, decouple at much larger densities and temperatures inside the remnant. Electron neutrino and positron absorption on neutrons increases substantially the electron fraction in the material, with a larger effect in hotter remnants and along the polar regions, where neutrino fluxes are more intense due to the lower optical depths [49, 125–129].

New degrees of freedom or new matter phases in the EOS at extreme densities ${\sim }3-5\rho _0$ can also impact the remnant dynamics and leave detectable imprints on the GW. Examples are matter models including hyperon production [92, 93] or zero-temperature models of phase transitions to quark-deconfined matter [94, 95]. In both cases, the EOS models soften at extreme densities thus favouring more compact remnants and their gravitational collapse. The impact of these processes on the dynamics depends on the densities at which the EOS softens (or stiffens). Postmerger GWs at kiloHertz frequencies carry, in principle, signatures of a rapid EOS softening (or stiffening) at postmerger densities. However, the unambiguous extraction of information from these detections will crucially depend on the (unknown) physics details and on the availability of theoretical models. For example, if the new matter phases impact the EOS weakly and/or at large densities $\rho \gtrsim 5\rho _0$ that are reached only during the remnant’s gravitational collapse, then no significant imprint in the GW is expected. In addition, the extraction of information on the EOS or NS properties from the kiloHertz spectrum requires the assumption of particular waveform models that depend on the EOS used in the simulations [75]. Examples of such models are those connecting the GW spectrum frequencies to the binary properties, and they are discussed next.

The dynamical phase described above lasts for about ${\sim }10{-}20$ milliseconds until the cores have completed their fusion or collapsed to a BH. During the core fusion, the remnant is a strongly deformed object with a pronounced bar-like deformation that powers a significant emission of GWs. The main postmerger GW signature is a short transient with a spectrum peaking at a few characteristic frequencies [19, 24–28, 91, 97, 130–133]. The main peak frequency is associated with twice the dynamical frequency of the remnant NS at early postmerger times $f_2\sim \varOmega /\pi$. It is important to note that $\varOmega$ evolves in time and that the GW spectrum is not discrete. The peaks are instead a consequence of the efficiency of the emission process: since the emission is very fast at early times, the spectrum is dominated by the broad peaks at the (approximately constant) frequencies right after merger [28, 74]. The GW postmerger spectrum can be robustly computed from short and nonexpensive simulations, thus has been studied in great detail. The characteristic peaks in the spectrum are often associated to hydrodynamical modes in the remnant, e.g. [18, 24, 80], and are thus often interpreted in analogy to linear perturbations of equilibrium NSs [134–136]. We refer to the literature above for detailed analysis of the characteristic postmerger frequencies and their association to the hydrodynamical modes in the remnant.

**Fig. 4:**
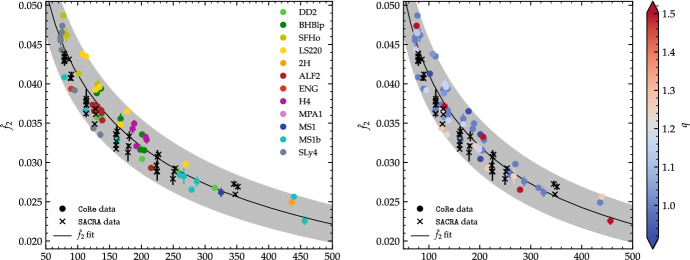
Phenomenological EOS-insensitive relation between the GW’s main peak postmerger frequency and the (modified) tidal parameter $\xi (\kappa ^\mathrm {T}_2,\nu )$ (Eq. (11)). Both panels show the same data. The round markers correspond to the simulations of the CoRe database. For those data the EOS variation is highlighted in colors in the left panel and the mass ratio variation in the right panel. The crosses correspond to the SACRA database that also refer to a large variation of EOS (although not highlighted in the graphics). Note that the frequency is the mass rescaled one in dimensionless units, $\hat{f}_{2}=Mf_{2}$. The fit is performed only on the CoRe data and the gray band represents the 90% confidence region. Figure adapted from [75]

The postmerger peak frequencies approximately correlate with the properties of the binary and to properties of the nonrotating NS equilibria constructed with the same EOS, e.g., [25–28, 137–140] (see also [141] for a review). EOS-insensitive phenomenological descriptions of the postmerger GW are thus possible. As an example, Fig. 4 shows a representation of the peak postmerger frequency in terms of $\kappa ^\mathrm {T}_2$ [28]. The basic idea behind this model is that the angular momentum available at merger determines the rotation $\varOmega$ of the bulk mass, and that the GWs are efficiently radiated in short time at this frequency. We stress again that the postmerger waveform is not formed by a set of discrete frequencies but rather the frequency evolves continuously in a nontrivial way, increasing (in a time-averaged way) in time as the remnant becomes more compact. EOS-insensitive relations are the base to construct simple analytical representations of the postmerger GW [26, 75, 142–144]. The use of these models to constrain matter at extreme densitites using kiloHertz GWs is explored in various works, e.g. [75, 137, 145, 146].

The GW luminosity depends strongly on the merger remnant, as illustrated by Fig. 1. For prompt collapse mergers the GW luminosity peaks are the largest and happen shortly after the moment of merger. Short-lived remnants have multiple peaks of comparable luminosity on a time scale of a few milliseconds postmerger [64]. These luminosity peaks correlate with the oscillations of the instantaneous GW frequency (see middle panel of Fig. 1) and correspond to the bounces of the NS cores. Long-lived (and stable) NS remnants are qualitatively similar to the short-lived ones but the GW emission is less intense due to the smaller compactness.

A main difference with respect to binary black holes is that the most luminous mergers do not correspond, in general, to those that radiate the largest amount of energy. The largest GW energies per unit mass are radiated by short-lived remnants over typical timescales of a few tens of milliseconds after the moment of merger [74]. This is because a bar-deformed remnant NS close to gravitational collapse is a very efficient emitter of GWs. The analysis of the energetics from the simulations indicates that about two times the energy emitted during the inspiral and merger can be emitted during the postmerger phase. This is shown for a representative BNS in Fig. 2: the binding energy at the moment of merger is $-E_b\sim 0.07$, while after the postmerger transient is $-E_b\sim 0.12-0.16$. While the merger energy and peak luminosity tightly correlate with $\kappa ^\mathrm {T}_2$, the total GW energy emitted by the remnant has a more complex behaviour. An absolute upper limit to the GW energy estimated by about one hundred simulations of the CoRe collaboration is [64]

19 $$\begin{aligned} E_{\mathrm{GW}} \lesssim 0.126\, \left( \frac{M}{2.8M_{\odot }}\right) ~M_{\odot }\mathrm{c}^2 \, . \end{aligned}$$

**Fig. 5:**
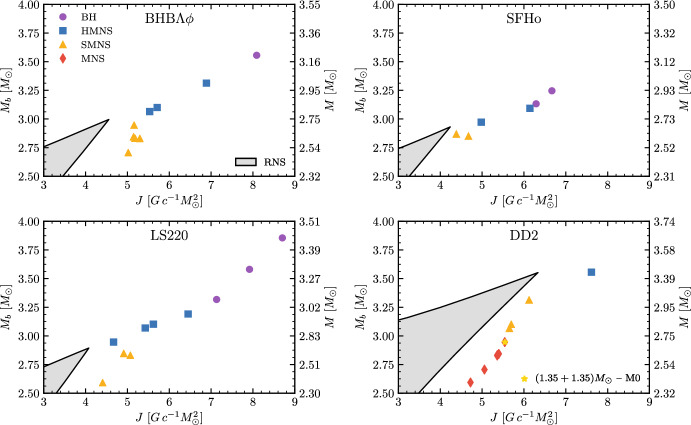
Diagrams of the rest-mass vs. angular momentum for representative merger remnants after the GW-dominated phase. The markers indicate remnants from fiducial mergers $M\sim 2.7-2.8M_{\odot }$, $q\sim 1$ and microphysical EOS. The gray region is the stability region of rigidly rotating equilibria constructed assuming zero temperature and neutrino-less beta equilibrium [147]. For a fixed *J* lower and upper boundaries of the shaded areas are set by the mass shedding and maximum mass limit, respectively. The tip of the shaded region marks the maximum baryonic mass configuration supported by each EOS in the case of rigid rotation. Figures from [31]

*Secular (Viscosity-driven) phase.* As the GW emission of energy and angular momentum backreacts on the fluid, it quickly damps nonaxisymmetric modes in the remnant that evolves towards axisymmetry. The GW-emission timescale estimated at the end of the dynamical phase is [31]

20 $$\begin{aligned} \tau _{\mathrm{GW}} = \dfrac{J}{{\dot{J}}_{\mathrm{GW}}}\gtrsim 0.5\, \mathrm{s} \, . \end{aligned}$$

At this point the dynamics become dominated by viscous and cooling processes.

The NS remnants that did not collapse to BHs have angular momenta significantly exceeding the mass-shedding limit for rigidly rotating NSs [31]. Figure 5 shows a diagram of the baryon mass $M_b$ and the angular momentum *J* of the remnant for a sample of remnants, and it compares them to the rigidly rotating zero-temperature and beta-equilibrated isolated NS equilibria (gray shaded region). The GW-driven phase in mergers’ remnants always ends on the right of the shaded areas; these remnants could be called “super-Keplerian”. Moreover, long-lived remnants have gravitational masses ${\sim }0.08\, M_{\odot }$ larger than the corresponding equilibrium models having the same baryonic (rest) mass, but zero temperature [31]. A key open question for future simulations is the evolution of these systems on timescales of hundreds of milliseconds to seconds postmerger.

The remnant evolution is determined by magnetohydrodynamics processes and neutrino cooling and heating that affect the NS rotation and its temperature. On the one hand, finite temperature and finite neutrino chemical potential contribute to ${\sim }10\%$ increase of the pressure in the NS core [30, 148]. Note that this is not sufficient to significantly alter the maximum nonrotating mass due to the degeneracy of matter above $\rho _0$. On the other hand, thermal support inflates the regions with subnuclear densities increasing the NS radius. For characteristic temperatures, the radius of a fiducial NS of mass $1.4M_{\odot }$ increases by about 20–40% (depending on the EOS) compared to the zero-temperature nonrotating case.

Rotational support also increases the maximum NS mass. For example, in the limiting case of rigid rotation at the Keplerian limit, the maximum NS mass is increased by ${\sim }20\%$ with respect to a nonrotating NS. Since this affects the whole star, the NS radius is typically increased by ${\sim } 40\%$, but at the same time the central density is decreased by a similar amount if one compares nonrotating and Keplerian NSs of identical masses. Interestingly, at temperatures reached in merger remnants, the maximum mass for a stable rigidly-rotating “hot” NS remnant is actually smaller than that for cold equilibria [148]. Rigidly-rotating NSs with temperature profiles similar to those found in simulations can support ${\sim }0.1\,M_\odot$ less baryonic mass than cold configurations. While it is unlikely that finite temperature and composition effects can stabilize a merger remnant against gravitational collapse, larger radii imply that the mass shedding limit is reached at lower angular frequencies. Hence, a NS remnant classified SMNS according to the cold EOS could actually collapse to a BH. Alternatively, it might be possible to form stable NS remnants with baryonic masses and thermodynamics profiles for which there is no rigidly-rotating equilibrium.

Magnetic fields also introduce additional pressure and can increase the maximum mass and the maximum velocity of a rigidly rotating isolated NS. However, the changes in maximum mass are moderate and up to 15–30% for extreme values of the magnetic field $B\sim 10^{18}$ G [149]. In merger remnants, magnetohydrodynamics instabilities and magnetic-field amplifications can lead to global-scale magnetic effects and angular momentum redistribution [17, 35, 150–153]. These instabilities operate on length scales of meters to centimeters, and it is presently impossible to perform fully-resolved, global merger simulations with realistic initial conditions. High-resolution simulations of mergers with magnetar-strength magnetic fields showed that the Kelvin–Helmholtz instability at merger could amplify the magnetic-field energy to up to 1% of the thermal energy [154]. Moreover, if turbulent stresses are modeled by an effective $\alpha$-viscosity, these simulations estimate $\alpha \simeq 0.01{-}0.02$ at $\rho \lesssim 10^{13}~\mathrm{g~cm^{-3}}$ (disc’s densities) and $\alpha \sim 10^{-4}{-}10^{-3}$ at higher densities [152]. Assuming the $\alpha -$viscosity model [155], the angular momentum redistribution in the remnant happens on a timescale [26]:

21 $$\begin{aligned} \tau _{\mathrm{visc}}\simeq & {} \alpha ^{-1}~R_{\mathrm{rem}}^2~\varOmega _{\mathrm{rem}}~c_s^{-2} \nonumber \\\simeq & {} 0.56~\mathrm{s} \left( \frac{\alpha }{0.001} \right) ^{-1} \left( \frac{R_{\mathrm{rem}}}{15\mathrm{km}} \right) ^2 \left( \frac{\varOmega _{\mathrm{rem}}}{10^4\mathrm{kHz}} \right) \left( \frac{c_s}{0.2c} \right) ^{-2} \, , \nonumber \\ \end{aligned}$$

where $\varOmega _{\mathrm{rem}}$ and $c_s$ are the remnant angular velocity and typical sound speed, respectively. Simulations including a prescription for treating viscosity in GR find that the remnant becomes more quickly axisymmetric, possibly reducing the postmerger GW emission [156, 157]. In particular, the turbulence induced by the magnetic field favours angular momentum redistribution and accelerates the collapse or significantly affects the remnant lifetime [26, 156]. The magnitudes of these effects depends on the particular value assumed for the $\alpha$-viscosity subgrid model. For example, the use of a turbulence model calibrated to the high-resolution MHD runs of [152], leads to significant changes to the subdominant features of the GW spectrum and to the ejecta [158]. However, neutrino effects on the ejecta are comparatively more relevant than magnetohydrodynamical turbulence.

The angular momentum redistribution in the remnant leads to characteristic rotational profiles with a local minimum at the center [37, 118, 159–162]. Since hydrodynamical and viscous effects counteract the gravitational instability of the core, the remnant’s core is expected to spin up and to reduce its compactness [33, 156]. This suggests that a super-Keplerian remnant evolving towards equilibrium must shed excess angular momentum. Because the angular momentum losses cannot be GW-driven (Eq. (20)) they must be driven by viscous effects on timescales of $\tau _{\mathrm{visc}}$ and other electromagnetic processes that can extract the rotational energy of the remnant, e.g. [163, 164]. These processes can very efficiently generate large outflows because the mass shedding limit moves to lower angular momenta with decreasing rest-mass $M_b$ [32, 33].

Simulating the timescales $\tau _{\mathrm{visc}}$ is challenging for ab-initio numerical simulations, so such a regime is currently explored in simplified setups (Newtonian gravity, axisymmetry, ad-hoc initial conditions, etc., see Sect. 6). Together with viscous processes, neutrino interactions are the other key process for the remnant evolution. The main effect is cooling, that operates on timescales up to $\tau _{\mathrm{cool}}\sim 2-3$ s [29, 55, 119, 165]. In addition, the excess of gravitational binding energy in the remnant found in NR simulations is likely radiated in the form of neutrinos. These conditions are analogous to those found in newly born NSs in core-collapse supernovae (e.g., [166–170]).

A possible outcome of the viscous evolution of a long-lived remnant is a rotating NS close to the mass shedding limit with spin periods $P_0 \lesssim 1\, \mathrm{ms}$. Comparing possible evolution scenarios to equilibrium sequences, it is possible to estimate [31]

22 $$\begin{aligned} P_0 = \left[ a \left( \frac{M_b}{1\, M_\odot } - 2.5\right) + b\right] \mathrm{ms}\ , \end{aligned}$$

with EOS-dependent coefficients $a\sim -(0.2{-}0.3)$ and $b\sim 1$. Note that the above estimate gives spin periods significantly smaller than those typically inferred for the progenitors of SGRB with extended emission in the context of the magnetar model, $P_0 \sim 10\, \mathrm{ms}$ [171, 172]. Gravitational-wave losses could however continue past the viscously-driven phase of the evolution and further spin down the remnant over a timescale of many seconds to minutes [171, 173]. The GW emission could be driven by secular instabilities in the remnant [8–12, 131, 138, 174–180] (see also [176] for a review), or by deformations due to a strong toroidal field [171]. For example, the GW luminosity of the one-armed instability during the first ${\sim }50\, \mathrm{ms}$ of the post-merger evolution is ${\sim }10^{51}\, \mathrm{erg}\, \mathrm{s}^{-1}$ and does not show strong evidence for decay [131, 179]. If the one-armed instability were to persist without damping, then it would remove all of the NS remnant rotational energy, which is ${\sim }10^{53}\, \mathrm{erg}$ [85], over a timescale of ${\sim }100\, \mathrm{s}$. This timescale is compatible with the spin-down timescale inferred from the magnetar model [171]. This GW signal from the one-armed instability could be detectable by LIGO-Virgo up to a distance of ${\sim }100\, \mathrm{Mpc}$ for optimally oriented sources [131].

**Fig. 6:**
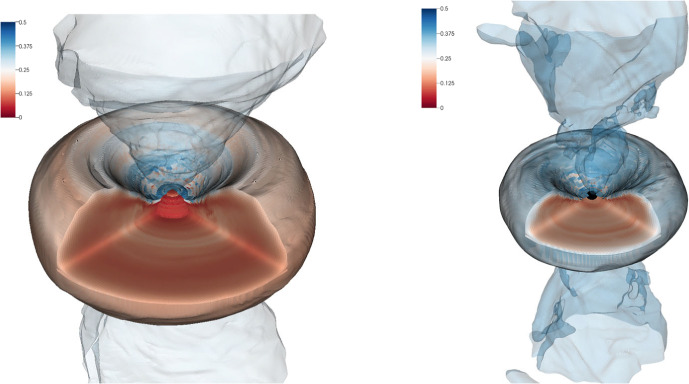
Example of discs around NS (left) or BH (right) remnants. The figure shows a 3D rendering of the electron fraction for equal-masses BNSs described by the DD2 (left) and SFHo (right). Both images have the same spatial scale and show the data in a box of size 750 km. The electron fraction is used to color the $10^7\,\text {g cm}^{-3}$ (semi-transparent) and the $10^{11}\,\text {g cm}^{-3}$ density isosurfaces. The $10^{13}\,\text {g cm}^{-3}$ isosurface is also shown for the DD2 model. The black surface in the SFHo model denotes the approximate location of the black hole horizon. The discs are fairly neutron rich in their bulk, irrespective of the remnant type (massive NS or black hole). The accretion disc coronae are irradiated by neutrinos and are less neutron rich. Figure reproduced with permission from [30], copyright by SIF / Springer

## Remnant discs

Following a common convention, the remnant disc is here defined as the baryon material either outside the BH’s apparent horizon or that with densities $\rho \lesssim 10^{13}\,\text {g cm}^{-3}$ around a NS remnant. The baryonic mass of the disc is computed in simulations as volume integrals of the conserved rest-mass density and it is referred to as $M_{\mathrm{disc}}$. Remnant discs are geometrically thick discs with typical aspect ratio $H/R \sim 1/3$ and mass between $0.001{-}0.2\ M_{\odot }{}$. The structure and composition of the remnant discs can significantly depend on the different formation mechanisms due to the different binary properties.

In the case of comparable mass mergers, the accretion disc is formed during and after the merger by the matter expelled by tidal torques and by the collision interface. Because of the different temperatures in the tidal tail (cold) and collisional interface (hot), the disc is initially highly non-uniform. As time evolves, the NS remnant continuously sheds mass and angular momentum into the disc with spiral density waves as described in Sect. 5, thus increasing the mass of the disc and generating mass outflows [31, 74] (see also Sect. 7). The continued action of shocks and spiral waves increases the entropy in the disc and eventually produces an approximately axisymmetric Keplerian disc characterized by a temperature profile that changes smoothly from $\sim 10\,$MeV (for $\rho \simeq 10^{13}\,\text {g cm}^{-3}$) down to ${\sim }0.1\,$MeV (for $\rho \simeq 10^{4}\,\text {g cm}^{-3}$). The electron fraction is reset by pair processes and the entropy per baryon varies between 3 and several 10’s of $k_{B}$/baryon [30]. In general, BH formation significantly affects the disc properties, as illustrated by Fig. 6. If the central object collapses to a BH, approximately half of the disk mass is swallowed inside the apparent horizon within a dynamical timescale, and the maximum density decreases to a few times $10^{12}$ $\text {g cm}^{-3}$. Discs around a BH remnant are in general more compact and achieve higher temperatures and entropies ($\varDelta s \simeq 2~{k_B/\mathrm{baryon}}$) than discs hosting a NS remnant.

**Fig. 7:**
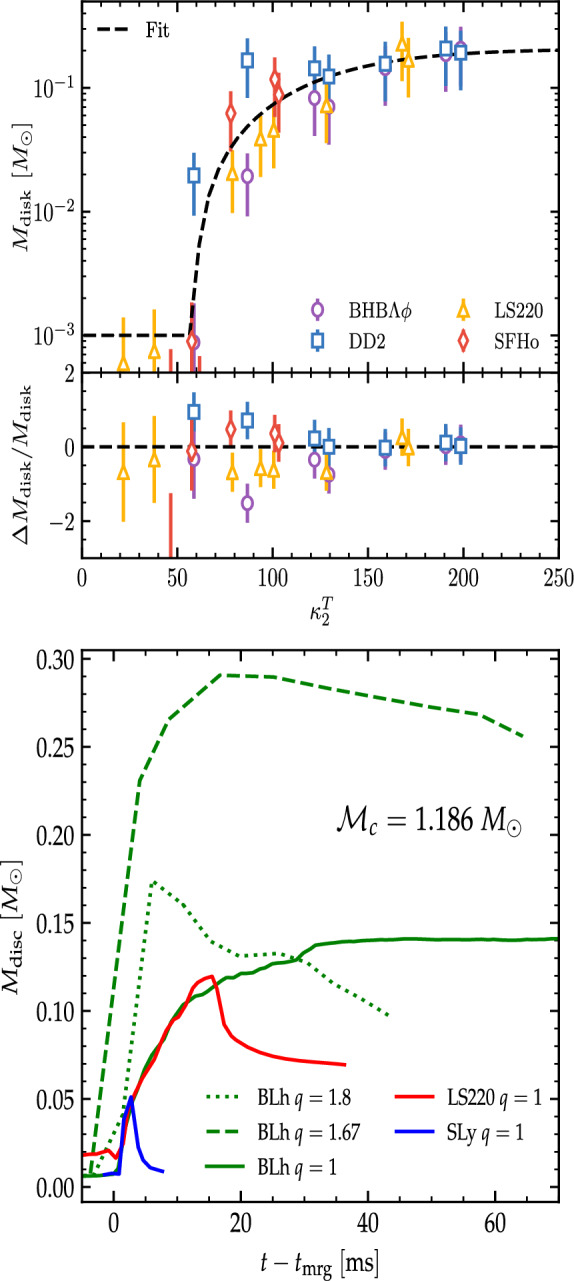
Disc masses as a function of the tidal parameter and disc mass evolution for representative cases. Top: The remnant disc mass of equal-mass mergers correlates with $\kappa ^\mathrm {T}_2$, the latter measuring the binary compactness. Small values $\kappa ^\mathrm {T}_2\lesssim \kappa _{\mathrm{pc}}$ correspond to prompt collapse mergers for which a disc with negligible mass forms. Figure adapted from [33]. The bottom panel shows the relative difference between the data and the fit. Bottom: Disc formation and early evolution for mergers with chirp mass $1.186M_{\odot }$. The $q=1$ SLy and LS220 are short-lived remnants collapsing to BH within 2 and 18 milliseconds respectively. The BLh $q=1,1.67$ are long-lived remnants, while the $q=1.8$ is an accretion induced prompt collapse. Figure adapted from [81]

Disc masses at formation are shown in the top panel of Fig. 7 as a function of the tidal parameter $\kappa ^\mathrm {T}_2$. Again, the choice of $\kappa ^\mathrm {T}_2$ for this plot is for correlating the disc with a measure of the binary compactness [Note however that the parameter is not a good choice for cases dominated by tidal distruption^8^]. The figure highlights that for $q\sim 1$ prompt collapse mergers do not form massive discs (cf. Eq. (16)), because the mechanism primarily responsible for the formation of the disc shuts off immediately in these cases, e.g., [37, 38, 40]. Short-lived and long-lived remnants have instead discs with initial masses ${\sim }0.2\,M_{\odot }{}$. Mergers of BNSs with mass ratios up to $q\sim 1.3$–1.4 however produce more massive discs than $q=1$ because of the larger centrifugal support and a partial tidal disruption of the companion NS [36–39, 97].

In high mass-ratio mergers with $q\gtrsim 1.5$ the companion NS is tidally disrupted and the disc is mainly formed by the tidal tail [81]. The latter is launched prior to merger and massive accretion discs are possible even if prompt BH formation occurs [34, 81]. The angular momentum of these discs can be ${\sim }60\%$ larger than that of discs around BHs resulting from equal-mass mergers. Moreover, due to the absence of strong compression and shocks, the discs formed in high mass-ratio mergers are initially colder and more neutron rich than those of comparable-mass mergers having the same chirp mass.

Examples of disc mass evolutions at early times from formation for different remnants are shown in the right panel of Fig. 7. The figure clearly shows the rapid accretion in case of equal-masses ($q\sim 1$) mergers and BH formation. Discs around NS remnants instead can also increase their mass over time as the remnant’s spiral waves propagate outwards. The accretion of discs around BHs formed in high-mass-ratio mergers is instead slower due to the larger disc’s extension and angular momentum. Here, however, accretion is further driven by the fallback of the tidal tail that perturbs the disc inwards [81].

The long-term evolution of these discs is key for electromagnetic emission, and studies in this direction are becoming more complete and detailed [32, 41–45, 51, 53, 183, 184]. However, the challenges related to the simulation of multiples scales and multiple physical processes have, so far, required the adoption of some simplifications. All of the published simulations either adopted somewhat artificial initial conditions (not derived from merger simulations), neglected important physical effects such as neutrino emission and absorption, assumed axisymmetry, or did not follow the evolution for sufficiently long times. Crucial questions are related to the development of ordered large-scale magnetic fields formed by dynamo processes and the interplay with neutrino interactions. Large-scale magnetic fields can power relativistic jets [109, 185–188] and drive mildly relativistic outflows [44, 53, 189]. Neutrinos emitted from the hottest and densest part of the remnant irradiate the low density part of the disk (and the expanding wind) thus increasing substantially the electron fraction in the material [183]. The larger effects are for hotter remnants and along the polar regions, where neutrino fluxes are more intense due to the lower optical depths [49, 123–129, 183]. The combined effect of magnetohydrodynamics and neutrino processes is likely to play an important role in the dynamics and should be further explored by future simulations [105, 188, 190].

## Mass ejecta

Since the ejection of neutron rich material happens at different stages of the merger dynamics, mass ejecta have multiple components with different properties, geometries and composition [191, 192].

*Dynamical ejecta.* Dynamical mass ejecta are launched during the merger process. A fraction of the material is launched by tidal torques around the moment of merger [46, 97, 126]; another fraction of matter is unbound from shocks generated after the moment of merger when the cores bounce [33, 47, 48, 127]. General-relativistic merger simulations indicate that the mass of the dynamical ejecta ranges from $10^{-4}\ M_{\odot }$ to $10^{-2}\ M_{\odot }$ and that it has characteristic velocities of $0.1{-}0.3$c [33, 47, 48, 127]. The tidal ejecta are neutron rich $Y_e \sim 0.1$ and cold, while the shocked ejecta are reprocessed to higher $Y_e$ by pair processes and neutrino irradiation from the NS remnant. The electron fraction in shocked ejecta can span a wide range of values, $Y_e\sim 0.1-0.4$, with the largest $Y_e$ obtained at high latitudes. If large-scale magnetic fields are present at the moment of merger, they could additionally boost the dynamical (shocked) ejecta with a viscous component [193].

**Fig. 8:**
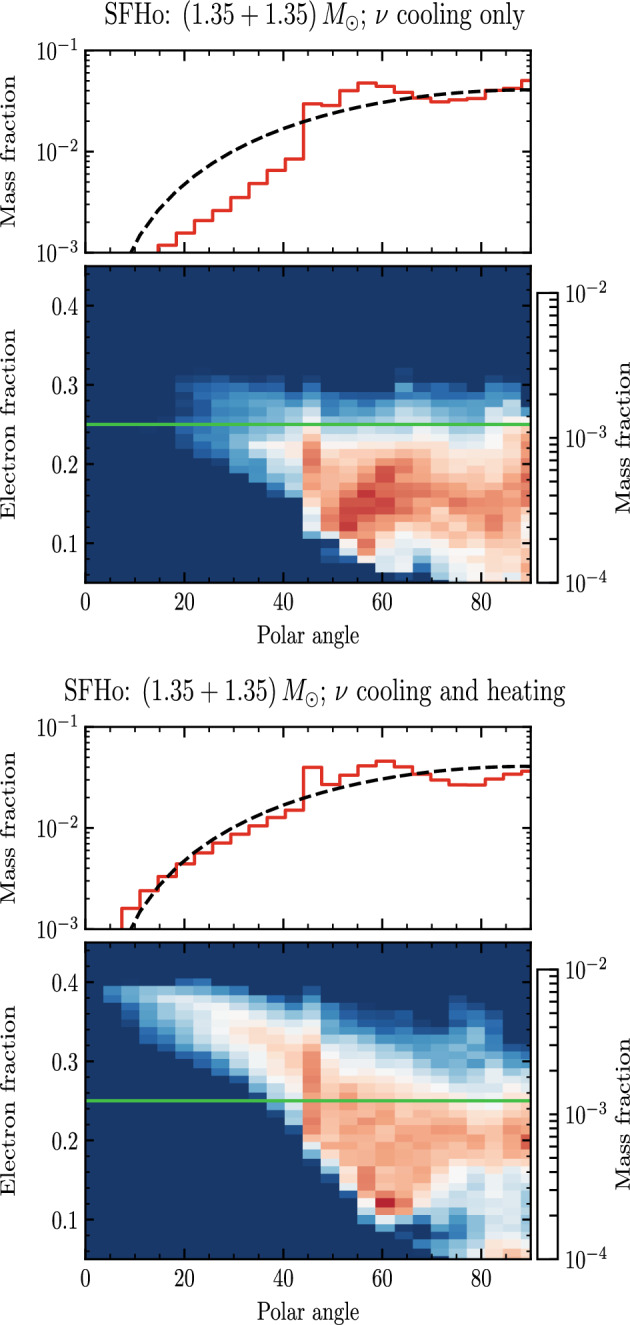
Dynamical ejecta mass and composition as a function of the polar angle $\theta$. The orbital plane is $\theta =90^\circ$. The top panel refers to a simulation with neutrino leakage only, the bottom panel to a simulation with neutrino leakage and the M0 transport scheme for free-streaming neutrinos. The dashed black line refers to a model distribution $Y_e(\theta )\sim \sin ^2\theta$. Figure adapted from [200]

For comparable-mass mergers, NR simulations indicate that the shocked component is typically a factor ten more massive than the tidal component. This is in contrast to early works that employed Newtonian gravity and in which the tidal component dominated the ejecta due to the weaker gravity and stiffer EOS employed in those simulations [46, 119, 165, 194–199]. A sample of about 130 NR simulations using microphysics EOS and approximate neutrino transport indicate that ejecta masses do not strongly correlate in a simple way with the properties of the binary [33]. The average mass is ${\sim } 2\times 10^{-3}\ M_{\odot }$ [33], the mass-averaged speed is about $\langle v_{\mathrm{dyn}}\rangle \sim 0.18$c (although some part of the ejecta can reach high-speeds up to $\sim 0.8$c [33, 48]), and the mass-averaged electron fraction is $\langle Y^{\mathrm{dyn}}_e\rangle \sim 0.17$. Neutrino absoprtion has a significant effect on the composition of dynamical ejecta, and some radiation transport scheme that includes neutrino absoprtion must be considered in the simulations for a realistic estimate of $Y_e$ [49, 200]. The dynamical ejecta properties vary with the polar angle [200]. The mass is launched about the orbital plane with a r.m.s. of ${\sim }35^\circ$; the highest velocities and electron fraction are obtained at high latitudes where the medium densities are lower and the neutrino fluxes are more intense. In particular, the electron fraction has a profile well approximated by $Y_e\sim \sin ^2\theta$, where $\theta$ is the polar angle with the axis normal to the orbital plane. As an example, Fig. 8 shows the distribution of mass and $Y_e$ in the polar angle, for two simulations that differ only in the neutrino transport scheme employed. If a leakage scheme is employed, thus only neutrino cooling is simulated, then the ejecta have no material with $Y_e>0.25$. If also neutrino absorption is simulated, then the $Y_e$ distribution extends to $Y_e\sim 0.4$ in the region $\theta <60^\circ$.

**Fig. 9:**
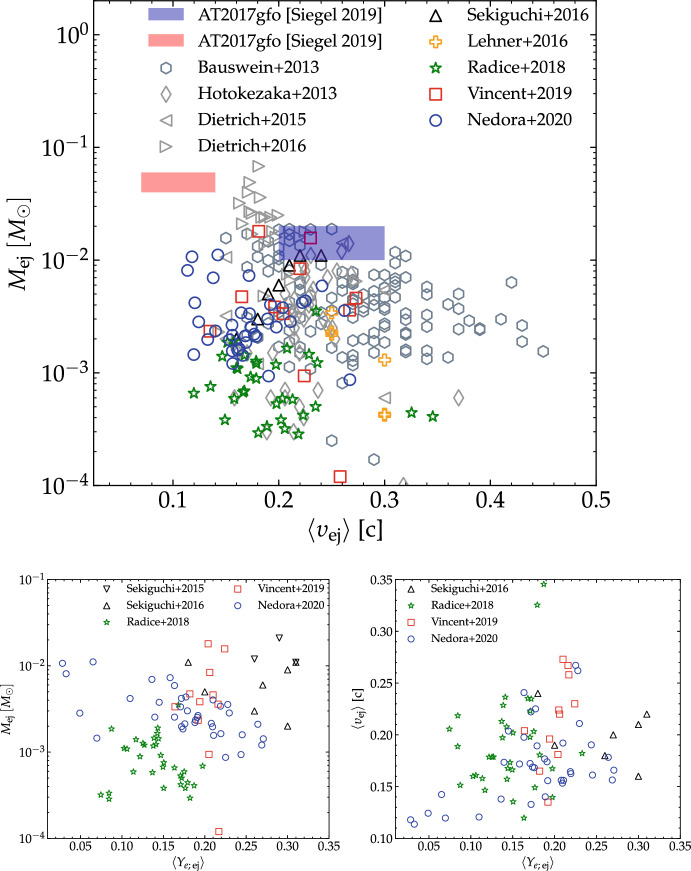
Summary of the dynamical ejecta properties (mass, mass-averaged velocity and electron fraction) as found by simulations with different physics input, different NS masses in ${\sim }1.2-1.5M_{\odot }$ and EOS. The datasets include: polytropic EOS data from [34, 47, 91, 205], microphysical EOS data with no neutrinos treatment from [48], microphysical EOS data with leakage scheme from [33, 132, 202], microphysical EOS data with M1 or leakage+M1 scheme from [127, 204], microphysical EOS data with leakage+M0 [30, 33, 81, 201, 206]. The filled blue and red patches are the expected values of ejecta mass and velocity for blue and red components of the kilonova AT2017gfo, compiled by [211] and based on [212]. Figure courtesy of V. Nedora

The dynamical ejecta data obtained by different groups with independent codes, for similar binaries and input physics are broadly consistent within a factor of two [33, 86, 120, 123, 132, 200–204]. Numerical errors can account for the difference in some cases, but for the highest resolutions simulated so far the numerical uncertainties are around 20-40% [33, 81, 86]. Figure 9 collects the dynamical ejecta properties for a representative sample obtained by various groups using different physics assumptions. In particular, it includes: (i) the piecewise-polytropic EOS runs of [34, 47, 91, 205], in which temperature effects are approximated by a $\varGamma$-law EOS and composition and weak effects are not simulated; (ii) the microphysical EOS data of [48] in which weak reactions are not simulated; (iii) the microphysical EOS data of [33, 132, 202] in which a leakage scheme is employed for neutrino cooling; (iv) the microphysical EOS data of [127, 204] in which a leakage+M1 scheme and a M1 gray scheme respectively are employed for the neutrino transport; (v) the microphysical EOS data of in which a leakage+M0 scheme are employed for the neutrino transport [30, 33, 81, 201, 206]. The largest differences in the computations reported in the literature are related to the use of different input physics. Microphysics and neutrino absorption have a significant impact on the dynamical ejecta properties [49, 200, 202, 204], as evident from Fig. 8. Microphysical EOS determine average velocities smaller than those computed using polytropic EOS, and distributed up to 0.3c. The inclusion of neutrino absorption results in larger average ejecta masses and electron fractions then those obtained with the leakage scheme. Simulations with polytropic EOS or without neutrino leakage, e.g. [47, 48, 205], give ejecta masses up to factor five larger than those obtained with simulations with microphysics and neutrino transport schemes, and in some cases average velocities up to $\langle v_{\mathrm{dyn}}\rangle \sim 0.3-0.4\,$c. Phenomenological fitting formulas of dynamical ejecta properties in terms of the binary parameters are presented in [33, 207, 208]. Note that the different fits are not fully consistent with each other; they depend on the particular datasets employed and some trends appear in the residuals. Simulations including microphysics and neutrino effects and spanning different chirp masses and mass ratios are required in order to quantify a clear dependence of the ejecta on the binary properties.

For highly asymmetric BNSs with $q\gtrsim 1.67$ the dynamical ejecta is instead dominated by the tidal component [81, 132, 202]. Here the ejecta is distributed more narrowly about the orbital plane and over a fraction of the azimuthal angle around its ejection angle with a crescent shape. Extreme mass asymmetry can boost the mass ejecta by up to a factor four with respect to the equal mass cases (for a fixed chirp mass). In this case, the average electron fraction reduces to ${\sim }0.11$, and the r.m.s. of the polar angle is ${\sim }5$–$15^\circ$ [81]. This is similar to what is observed in black-hole–neutron-star binaries, [209].

In asymmetric mergers of rotating NSs with spin aligned to the orbital angular momentum, the dynamical ejecta mass can increase due to the larger angular momentum of fluid elements in the tidal tail [91]. However, for equal-mass mergers the ejecta mass can decrease for large aligned spins [91, 210] because at the moment of merger the system is more bounded (smaller $j_{\mathrm{mrg}}$ and more negative $E_b^{\mathrm{mrg}}$ as aligned-spin-orbit interaction is repulsive) and less material is unbound from the core shock. Overall, spin effects are sub-dominant with respect to mass ratio effects [91].

*Secular ejecta.* Another type of ejecta is the secular winds originating from the remnant [33, 41–43, 45, 50–53, 124, 213]. Long-term Newtonian simulations of neutrino-cooled accretion disks indicate that $10{-}40\%$ of the remnant disk can unbind over a timescale of a few seconds. Since remnant discs in mergers have masses up to ${\sim }0.2\ M_{\odot }$, winds are likely to constitute the bulk of the ejecta (if present). These secular ejecta can originate from different physical mechanisms.

Neutrinos from the remnant and the disc drive a wind of material with $Y_e\sim 0.3$ and can unbind ${\lesssim }10^{-3}\ M_{\odot }$ [43, 124, 184, 213]. The neutrino wind originates on the disc edge, close to the neutrinosphere, and above the remnant where baryon pollution is minimal. Note that a precise prediction of properties of polar ejecta is presently beyond the possibilities of neutrinos schemes employed in ab-initio NR simulations [43, 45, 184, 214].

Long-term NR simulations have shown that, if the merger outcome is a NS remnant, the spiral density waves propagaing from the remant into the disc can trigger a massive and fast wind [201]. These ejecta start after the moment of merger and operate on timescales longer than the dynamical ejecta. Their origin is purely hydrodynamical but viscosity and neutrino transport influence the angular momentum transported by the spiral waves and their composition. The spiral wind can have a mass up to ${\sim }10^{-2}M_{\odot }$ and velocities $\lesssim 0.2$ c. The ejected material has electron fraction mostly distributed above $Y_e\sim 0.25$ being partially reprocessed by hydrodynamic shocks in the expanding arms.

Angular momentum transport due to viscous processes causes the disc to spread outwards. Once the accretion rate drops below a critical threshold, neutrino cooling becomes ineffective and the disc thermally expands [41, 50, 110]. At this point, recombination of nucleons into alpha particles provides sufficient energy to unbind ${\sim }10{-}20\%$ of the disc. The nuclear binding energy liberated in the process is ${\simeq }8.8$ MeV/nucleon. Because the disc material starts to recombine where the nuclear energy equals the gravitational binding energy, a characteristic cylindrical radius $\varpi ^*$ at which the wind starts is [41],

23 $$\begin{aligned} \frac{G M_{\mathrm{disc}} m_b}{\varpi ^*} \simeq 8.8\, \mathrm{MeV}\ , \end{aligned}$$

where $m_b$ is the baryon mass. These disc ejecta can be massive and are launched around the equatorial (orbital) plane with characteristic velocities ${\sim }0.1$ c [41, 51, 215]. Magnetohydrodynamics effects can enhance the secular masses and asymptotic velocities and boost the disc ejection fraction to up to ${\sim }40\%$ [52, 53]. For long-lived remnants, the composition of the secular ejecta depends sensitively on the lifetime of the remnant due to neutrino irradiation [33, 51].

From the above discussion it should be clear that several properties of the ejecta (and thus of the kilonova) depend sensitively on the remnant, although these dependencies are not fully quantified yet. This is further indicated by the fact that some of the broad features of synthetic kilonova light curves computed from fiducial NR data show a correlation with the tidal parameter ${\tilde{\varLambda }}$ (and hence the merger outcome) [33].

We finally mention the key elements connecting the ejecta and the kilonova emission. For a complete discussion see [63, 192]. The key quantity determining r-process nucleosynthesis in the ejecta is the electron fraction $Y_e$ [192, 216]. If $Y_e \lesssim 0.2$, then the ejecta produce second and third r-process peak elements with relative abundances close to solar ones. If $Y_e \gtrsim 0.3$, then the material is not sufficiently neutron rich to produce lanthanides but first r-process peak elements are produced. A sharp trasition between these two regimes is marked by $Y_e \simeq 0.25$. The $Y_e$ also determines the photon opacity in the material [61, 62], drastically altering the timescale and the effective blackbody temperature of the kilonova emission [63]. High-$Y_e$ ejecta power kilonovae peaking in the UV/optical bands within a few hours of the merger (blue), while low-$Y_e$ ejecta power kilonovae peaking in the infrared over a timescale of several days (red).

## Conclusion

It is useful to summarize by focusing on the concrete examples of the two BNS events observed so far, GW170817 [1–3, 217] and GW190425 [14].

The source of GW170817 has mass $M\simeq 2.73-2.77M_{\odot }$ and mass ratio up to $q=1.37$ (1.89) depending on the low (high) spin prior utilized in the GW analysis [1–3]. The merger was not observed but the merger frequency can be accurately predicted from the probability distribution of $\tilde{\varLambda }$ using the NR fits discussed in Sect. 2. One finds that the (broad) distribution of ${\tilde{\varLambda }}$ translates into $f_\mathrm {mrg}=1719^{+163}_{-214}$ Hz [75]. Combining the GW170817 data with the prompt collapse models of Sect. 3, it is possible to rigorously predict via a Bayesian analysis that the probability of prompt BH formation is ${\sim }50-70\%$. However, if the constraint on the maximum mass $M>1.97M_{\odot }$ from pulsar observations is imposed, the probability significantly decreases below $10\%$. Hence, prompt collapse in GW170817 is largely disfavoured by the GW analysis [84].

A NS remnant would have emitted GWs at the characteristic frequency $f_2=2932^{+337}_{-409}$ Hz, that can be again estimated from the ${\tilde{\varLambda }}$ posteriors together with the peak GW luminosity [64, 75]. A sufficiently sensitive network of GW antennas could have detected the postmerger GW at $f_2$ with a peak luminosity larger than $10^{55}$ erg/s. These frequencies and luminosities might be accessible by improving the design sensitivity of current ground-based GW detectors by a factor two-to-three or with next-generation detectors [146, 218–220].

The NR-based GW analysis of the prompt collapse supports the mainstream interpretation of the electromagnetic counterparts that suggests the formation of a short-lived NS remnant [40, 85–88, 221]. AT2017gfo, the kilonova counterpart of GW170817, has both a blue and a red component, thus suggesting that the ejecta had a broad range of compositions with at least a fraction being free of lanthanides. A fit of AT2017gfo light curves to a semianalytical two-components spherical kilonova model indicates the lanthanide poor (rich) blue (red) component has mass $2.5\times 10^{-2}\, M_{\odot }$ ($5.0\times 10^{-2}\, M_{\odot }$) and velocity 0.27c (0.15c) [212, 222] (see also Fig. 9). Similar results are obtained using more sophisticated 1D simulations of radiation transport along spherical shells of mass ejecta [223, 224]. The estimated masses are larger than those predicted from NR for the dynamical ejecta and the estimated velocities for the blue component are smaller than those expected for disc winds [215]. Note that in Fig. 9 the BNS models fitting the blue component have soft EOS and masses significantly lower than those of GW170817. Kilonova models with multiple components help in resolving the tension [200] because the faster dynamical ejecta can be irradiated by the underlying disc, thus sustaining the emission [42, 225, 226]. Also, spiral-wave winds [201] and/or highly magnetized winds [53, 188, 189] might contribute in filling the gap.

Within this picture, prompt collapse can be tentatively excluded by the observation of the blue kilonova. Under the assumption of an equal-mass merger, only a small quantity of shock-heated or disk wind ejecta would be present in this case and it would be inconsistent with the ${\sim }10^{-2}M_{\odot }$ inferred from the data [85]. A long-lived remnant could be excluded based on the estimated kinetic energy of the observed kilonova and SGRB afterglow, that are too low for the energy reservoir of a NS remnant at the mass shedding limit. Note that alternative scenarios based on the interaction between a relativistic jet and the ejecta exist [227–229], but they are disfavoured due to the insufficient deposition of thermal energy in the ejecta [230].

Under the assumption that the merger remnant was a short-lived NS, the NR models described in Sect. 3 and basic arguments led to estimates of $M_\mathrm {max}^\mathrm {TOV}\lesssim 2.1-2.3M_{\odot }$ [85–88]. Further, using empirical relations between NS radii and the threshold mass $M_\mathrm {pc}$ for prompt collapse it is possible to tentatively rule out EOS predicting minimal NS radii ${<}10$ km and radii at $1.6M_{\odot }$ $\lesssim 11$ km [221]. Combining the GW data and the phenomenological fit of the disc mass in Fig. 7 also leads to a possible lower bound on the tidal parameter and thus a stronger constraint on the tidal parameter $300\lesssim {\tilde{\varLambda }}\lesssim 800$ [40, 129].

GW190425 is associated to the heaviest BNS source known to date with $M\simeq 3.2$–$3.7M_{\odot }$ [14]. The mass ratio of GW190425 can be as high as $q\sim 1.25$ ($q\sim 2.5$) for low (high) spin priors. Using the NR prompt collapse models presented in Sect. 3, it is possible to estimated that the probability for the remnant of GW190425 to have collapsed promptly to a BH is ${\sim }97\%$ [14]. For an equal mass merger ($q\sim 1$), a prompt collapse does not form a significant disc as discussed in Sect. 6, and thus no bright electromagnetic counterparts would be expected from this event, e.g. [231]. However, the conclusions would be different in the scenario that GW190425 was produced by an asymmetric binary with $q\gtrsim 1.6$. For large mass ratios, the prompt collapse threshold significantly decreases and massive neutron-rich discs are likely [81]. One the one hand, the prompt collapse to BH outcome is strenghtened in the $q\gtrsim 1.6$ scenario. On the other hand, a bright and temporally extended red kilonova, similar to the one expected for BH-NS binaries, would have been an expected counterpart [15, 31, 81].

To conclude, future science with BNS merger observations will crucially depend on the quantitative characterization of the merger outcome. While numerical-relativity efforts towards physically realistic and quantitative models for multimessenger analysis are ongoing, the interplay between theory, simulations and observations appears necessary to guide these efforts.

## Appendix A: Numerical-relativity methods

Numerical-relativity simulations are based on the 3+1 formalism of general relativity [232–234]. This appendix schematically summarizes the main physical effects and techniques implemented in current state-of-art simulations.

- *Initial data* for circular merger simulation are prepared by solving the constraint equations of 3+1 general relativity in presence of a helical Killing vector and under the assumption of a conformally flat metric [235]. The EOS used for the initial data are polytropes or constructed from the minimum temperature slice of the EOS table employed for the evolution assuming neutrino-less beta-equilibrium. Consistent initial data for circular mergers with NSs with spin are constructed with an extension of the formalism that is suitable for a constant rotation velocity of the NS [236, 237].
- *The Einstein equations* are then solved with free-evolution schemes like BSSNOK [238–240] or Z4c [241–244] based on the conformal decomposition of the metric fields. The latter scheme (and variation on the original proposal [245–247]) incorporates improved constraints propagation and damping properties with respect to BSSNOK and is thus preferable to BSSNOK in nonvacuum spacetimes. Neutron star spacetime evolutions are also performed with the generalized harmonic scheme [248, 249].
- *Gauge conditions* are chosen as 1+log and Gamma-driver shift similarly to binary black hole simulations [250–254]. These conditions handle the singularities’ formation and movement as moving punctures [255–257].
- *General relativistic magnetohydrodynamics* is formulated in conservative form [258]. Finite volume methods are typically employed to solve the hydrodynamics. High-order reconstructions or shock-capturing finite diffencing schemes proved to be important for waveform modeling [77, 259, 260]. Magnetohydrodynamics is typically handled using constrained-transport schemes to control the magnetic-field divergence [17, 261–268]. The use of the vector potential [264] combined with the Lorentz gauge helps improving numerical stability when using structured meshes [266]. Another method employed to control the magnetic-field divergence in some simulations is divergence cleaning [269–271]. Nonideal (resistive) magnetohydrodynamics schemes have been formulated in general relativity although their applications to mergers have been limited to date [272–274].
- Approximate *neutrino transport schemes* are based on the moment formalism [275, 276] and/or the leakage scheme [165, 271, 277, 278]. In the M1 scheme, the moments representation of the Boltzmann equation is truncated at the second moment and a closure interpolating between the thin and thick regime is imposed to the equations. The compact binaries simulations performed so far with this scheme are performed in the gray regime [122, 214, 279]. The leakage scheme is an approximation to the transport problem that accounts for changes to the lepton number and for the loss of energy due to the emission of neutrinos. Momentum transport and diffusion effects are not taken into account by the leakage, but free-streaming neutrinos can be additionally treated by combining the leakage with the M1 closure scheme [127] or with the M0 scheme [33]. The latter is a simplified but computationally efficient scheme free of the radiation shock artifact that plagues the M1 scheme [214].
- *Equations of state* models simulated so far include Skyrme models with finite-temperature and composition dependency, e.g., the LS220 [280] and the SLy4 [281, 282]; relativistic mean field models [283] with temperature and composition dependencies like the DD2 [284, 285] and the SFHo [286]; and Brueckner–Hartree–Fock extensions to finite temperature like the BLh [287]. Softening effects at extreme densities have been simulated with EOS with $\varLambda$-hyperons like the Shen H. et al. [288] and the BHB$\varLambda \phi$ [289], or with quark-deconfinement transitions implemented by relativistic mean field models, e.g., [290–293]. Large samples of piecewise polytropic EOS [294] or cold/beta-equilibrated microphysical with a thermal pressure contribution given by a $\varGamma$-law have been simulated by various groups. Most of these EOS are compatible with present nuclear constraints and the cold, neutrino-less $\beta$-equilibrated matter predicts NS maximum masses and radii within the range allowed by current astrophysical constraints, including the recent GW constraints.
- *General-relativistic viscous hydrodynamics* schemes have been developed recently. One method is the general-relativistic large eddy simulations method (GRLES) [156]. Another method is based on a simplified Israel–Stewart formalism of general-relativistic shear-viscous hydrodynamics [157, 295]. Both approaches simulate turbulent viscosity by specifying an effective shear parameter proportional to the sound speed, $\nu \propto c_s$, that sets the intensity of the turbulence.
- The *computational domain* is typically covered by a structured grid composed of Cartesian overlapping domains (box-in-box) with 2:1 mesh refinement between parent and child. Evolutions are performed with method of lines and the Berger–Oliger algorithm with sub-cycling in time and refluxing [296, 297]. The computational domain covers from the interior of the stars to the radiation zone, with the possibility of moving some of the Cartesian boxes to follow the orbital motion. An outer spherical grid composed of multi-patches is sometimes used to extend the radiation zone [298, 299]. Spherical grids are being explored and could help for long-term simulations of the postmerger phase [247].
- *Gravitational waves* are extracted on coordinate spheres at large radii using metric (Regge–Wheeler–Zerilli) or curvature (Newman–Penrose) perturbation theory of spherical or axisymmetric spacetimes [300–302].
- *Mass ejecta* are computed as those fluid elements that satisfy either the geodesic criterion, $-u_t > 1$ where $u^\mu$ is the fluid’s 4-velocity, or the Bernoulli criterion, $-hu_t > 1$ where $h\ge 1$ is the enthalpy, on large-radii extraction spheres. Both criteria are approximate and apply to stationary spacetimes. The former criterion assumes the ejecta’s fluid elements are on ballistic trajectories and neglects the fluid’s pressure; the latter is more appropriate for steady flow. The geodesic criterion is usually adopted for the fast dynamical ejecta while the Bernoulli one is employed for the winds.
- *Black-hole horizons* and BH properties are computed using apparent horizons [303].

Publicly available NR datasets from merger simulations exist and will be significantly growing in the next years. Gravitational waveforms for hundreds of configurations have been released by the CoRe collaboration [82], the MPI/Kyoto group [304] and the SXS collaboration [305] on their websites^9^ Ejecta data from the CoRe collaboration [33] are available on Zenodo [306]. There exists a Zenodo community called *NRGW open data* that hosts a collection of datasets from numerical relativity and gravitational waves modeling papers:

https://zenodo.org/communities/nrgw-opendata/

Data upload and download are open and welcome.

## Footnotes

1 A BH-NS source for GW has not been firmly excluded, e.g. [15]

2 This review reflects the views of the author who aimed at a brief but updated overview avoiding a more complete historical perspective.

3 There exist also spin multipole moments and gravitomagnetic tidal moments. The discussion here is simplified.

4 The dependency on the orbital separation follows immediately from the Lagrangian at leading order $L\approx +\mu _2 G_{ab}G^{ab}$ and the general property $G_L\propto \partial _L u \propto r^{\ell +1}$.

5 A more precise and formal argument is discussed in [75, 78].

6 The corresponding value in ${\tilde{\varLambda }}$ is

7 Note, however, that the nomenclature is retained in some of the presented figures.

8 An extreme case is for example the disc mass in black-hole–neutron star binaries, that does not show strong correlation with $\kappa ^\mathrm {T}_2$, [181, 182]. See discussion in [75].
